# Impact of climate change on river water temperature and dissolved oxygen: Indian riverine thermal regimes

**DOI:** 10.1038/s41598-022-12996-7

**Published:** 2022-06-02

**Authors:** M. Rajesh, S. Rehana

**Affiliations:** Hydroclimatic Research Group, Lab for Spatial Informatics, International Institute of Information Technology Hyderabad, Hyderabad, India

**Keywords:** Climate change, Environmental impact, Hydrology, Civil engineering

## Abstract

The impact of climate change on the oxygen saturation content of the world’s surface waters is a significant topic for future water quality in a warming environment. While increasing river water temperatures (RWTs) with climate change signals have been the subject of several recent research, how climate change affects Dissolved Oxygen (DO) saturation levels have not been intensively studied. This study examined the direct effect of rising RWTs on saturated DO concentrations. For this, a hybrid deep learning model using Long Short-Term Memory integrated with k-nearest neighbor bootstrap resampling algorithm is developed for RWT prediction addressing sparse spatiotemporal RWT data for seven major polluted river catchments of India at a monthly scale. The summer RWT increase for Tunga-Bhadra, Sabarmati, Musi, Ganga, and Narmada basins are predicted as 3.1, 3.8, 5.8, 7.3, 7.8 °C, respectively, for 2071–2100 with ensemble of NASA Earth Exchange Global Daily Downscaled Projections of air temperature with Representative Concentration Pathway 8.5 scenario. The RWT increases up to7 °C for summer, reaching close to 35 °C, and decreases DO saturation capacity by 2–12% for 2071–2100. Overall, for every 1 °C RWT increase, there will be about 2.3% decrease in DO saturation level concentrations over Indian catchments under climate signals.

## Introduction

River water quality parameters such as River Water Temperature (RWT), and Dissolved Oxygen (DO) forms vital signs for defining the health of a river water body’s ecosystem^1^. Global warming climates have also shown an adverse impact on RWT under intensification various climatological defining variables, majorly Air Temperature (AT)^2–4^. Intensification of RWT will have adverse impacts in terms of a decrease of river DO saturation levels, where most of the river water quality standards are defined based on such saturation levels^3^. Precisely, saturation DO is a prominent indicator of river water quality and is considered a standard measure to define the pollutant extent^5^. The influence of climate change on DO in relation to RWT can lead to water quality degradation and ecological distortion^6–11^. RWT is inversely related to DO concentration that every change in RWT affects the river's ability to self-purify by lowering the amount of oxygen that can be dissolved and utilized for biodegradation^12–14^. Hence, climate change impacts on RWT and saturation oxygen content are prominent in understanding the projected river water quality and possible alterations in quality standards under climate change warming signals.

Water quality modeling studies predicted depletion of DO under streamflow, RWT, and land use changes for various basins globally^1,7,15–19^. Such studies modeled RWT based on regression models^18^ and process-based stream temperature models^15,16^ and river water quality models such as QUAL2K^17,19^. However, such studies are basin or river stretch specific, data intensive, and limits application for data sparse and ungauged locations with an emphasis on simulated DO levels in response to streamflow, RWT, and land use^16–19^. However, DO saturation level, which serves as a baseline to measure oxygen-based water quality by determining the oxygen concentration of unpolluted water depending on RWT, salinity, and oxygen partial pressure^1^ and is prominent in defining the maximum permissible limits and standards for various river usages^20,21^, has not been assessed under climate change. Specifically, while some recent studies have looked at how climate change affects RWTs, the question of how climate change affects saturation DO have yet to be answered. More specifically, the direct integration of RWT predictions in the assessment of DO saturation concentration levels under climate change signals has not been quantified. Therefore, the present study aims to quantify the projected changes in DO saturation levels under RWT projections using the state-of-the-art Global Climate Model (GCM) projections. Furthermore, saturation DO is generally considered a desirable level of DO by the Pollution Control Boards (PCBs) in Waste Load Allocation Models (WLAM) for river water quality management^22^. Therefore, the study of climate change impacts on saturation DO levels can provide prominent insights for defining/alterations of the water quality standards under climate signals.

Climate change has been demonstrated to have an impact on the relationship between RWT and DO concentrations in tropical rivers^7^. Tropical rivers receive more solar radiation and have higher RWTs^23^. For example, Indian tropical river systems experience the highest RWTs during low flow periods of non-monsoon and summer months^19,24^. Seasonality plays a vital role in the Indian river systems as maintaining flows in the summer season is a challenge leading to water quality deterioration. To this end, the assessment of DO saturation rates with respect to RWT is of much relevance for Indian river systems due to minimum flows and higher temperatures during non-monsoon seasons.

Accurate estimation of RWT is prominent and can be estimated based on thermal advection–dispersion models^25^, equilibrium temperature-based models^26^, statistical or machine learning (ML) models^27^, and hybrid models^28^. Unlike process-based models, ML models do not require many input variables, which are unavailable for many ungauged river systems and have been widely used as robust in RWT modeling in recent years^29^. In this context, regression models^18,27,30–34^, classical ML models^35,36^, Artificial Neural Networks (ANN)^37–45^, has proven to be a viable technique for RWT forecasting. Most Indian River systems are burdened with data limitations and form a significant challenge for implementing process-based RWT models and promotes to implement regression or ML based approaches to predict RWT^35^. Therefore, given the limitations over data availability for Indian river systems, the present study stresses on use of ML based prediction algorithms that can address the data availability limitations. In this study, we used Long short-term memory (LSTM) model coupling with the k-nearest neighbor (k-NN) bootstrap resampling simulation technique (kNN-LSTM) to achieve a better prediction of RWT under data limitations for seven major Indian catchments with monthly RWT data. In summary, the objective of the study is to calculate the impacts of climate change on riverine thermal processes in India and possible variability in DO saturation levels with respect to RWT by using the kNN-LSTM model addressing sparse spatiotemporal RWT data forced with state-of-the-art climate change projections. The study evaluated the effect of climate change on DO saturation with respect to seasonal RWTs with an ensemble of 21 General Circulation Models (GCMs) using Representative Concentration Pathway (RCP) 8.5 scenario dataset output downscaled from the National Aeronautics Space Administration (NASA) Earth Exchange Global Daily Downscaled Projections (NEX-GDDP) dataset. The present study considered seven majorly polluted catchments of India^46,47^ with various physiographic features to analyze climate change impacts on saturated DO with respect to predicted RWT using kNN-LSTM based ML model using NEX-GDDP projections.

## Methods

### kNN-LSTM model

The present study considered the most widely known RNN architecture of LSTM to predict the RWT due to the superiority of using backpropagation through time and overcoming the vanishing gradient problem, and capable of learning long-term dependencies^48–51^. The LSTM consists of different memory blocks called cells. Each memory cell has an input gate, an output gate, and an internal state that feeds back into itself unaffected over time steps, which learns when it’s time to forget about prior hidden states when to update hidden states given new data and be used to learn complex temporal sequences. These memories in LSTMs are called cells. This study used the ensemble of k-NN bootstrap resampling algorithm to simulate the data from historical records based on Raseman et al.^52^ and LSTM model (kNN-LSTM) for monthly RWT prediction at the seven catchment sites of India with sufficient tests of performance measures of a model. For future RWT projections, RCP 8.5 scenario down-scaled projections of AT data were fed into the kNN-LSTM monthly prediction model. To train the kNN-LSTM model, the current AT and previous month time-lag of both AT and water temperature of the k-NN algorithm-based data as predictors for seven catchments of India at monthly timescale. The first month’s water temperature is calculated based on the catchment mean from the historical record. The prediction of subsequent months proceeds as follows:

1 $$ T_{t + 1}^{w} = f\left( {T_{t + 1}^{a} , T_{t}^{a} , T_{t}^{w} } \right) $$

where $$T_{t + 1}^{w}$$ is the future RWT prediction at time *t* + *1* month; *f* is a non-linear function which is generated by the kNN-LSTM monthly model; $$T_{t + 1}^{a}$$ is the future AT at time *t* + *1* month; $$ T_{t}^{a} $$ is future AT at time *t* month; $$ T_{t}^{w}$$ is the predicted water temperature value at time *t* month.

**Figure 1:**
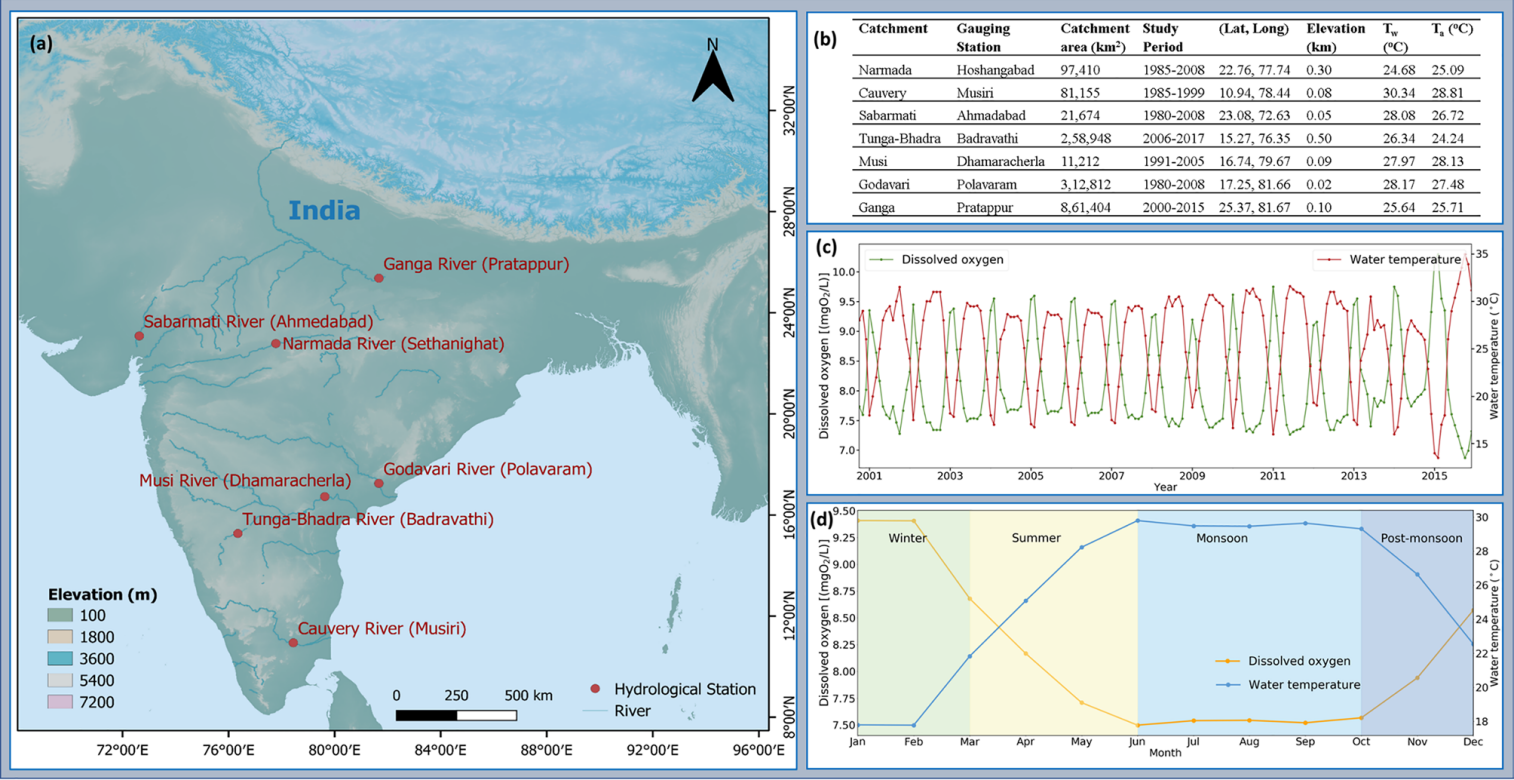
(**a**) Location map of study sites in India, (**b**) summarized all catchments and gauging station information in tabular form, (**c**) time series of monthly dissolved oxygen concentration (mgO_2_/L) and water temperature (°C) for the period 2001–2015 at Ganga catchment, and (**d**) monthly mean dissolved oxygen concentration (mgO_2_/L) and water temperature (°C) based on 14 years average at Ganga catchment for the period 2001–2015. The map was created using QGIS v3.4.14 (https://qgis.org), Python v3.7.4 (https://www.python.org), and post-processed with PowerPoint v2018 (https://microsoft.com).

For the analyses, we focused on the catchment’s observed data periods (Fig. 1b) and future periods 2021–2050 and 2071–2100, followed by the 30 years for a climatological standard normal^53^.

### Oxygen saturation

Waters with concentrations below saturation are called “deficit” whereas those with concentrations exceeding saturation are called “supersaturated”. As a result, the oxygen saturation concentration serves as the baseline for any endeavor to measure oxygen-based water quality by determining the oxygen concentration of unpolluted water^1^. The saturated DO concentration depends on the temperature, salinity of water, and oxygen partial pressure. Saturated DO concentration is influenced by these elements, as indicated by^54^

2 $$ o_{s} = \omega_{k} \cdot \omega_{s} \cdot e^{{\ln o_{sf} \left( T \right)}} $$

where $o_{s}$ = saturated DO concentration (mgO_2_/L), $\omega_{k} , \omega_{s}$ = elevation above sea level (dimensionless), and salinity (dimensionless) respectively, and $o_{sf}$ = the saturated DO concentration of sea-level freshwater (mgO_2_/L). The following are the individual impacts of temperature, salinity, and elevation.

#### Temperature, *T* (°C)

The saturated oxygen of fresh water at sea level is estimated by evaluating the exponent of the exponential function of Eq. (2) with^54^

3 $$ \begin{aligned} \ln o_{sf} \left( T \right) = & - 139.34411 + \frac{{1.575701 \times 10^{5} }}{{T_{abs} }} - \frac{{6.642308 \times 10^{7} }}{{T_{abs}^{2} }} \\ & + \frac{{1.243800 \times 10^{10} }}{{T_{abs}^{3} }} - \frac{{8.621949 \times 10^{11} }}{{T_{abs}^{4} }} \\ \end{aligned} $$

where $T_{abs}$ = absolute temperature in kelvin.

#### Salinity, *S* (ppt)

The oxygen saturation of seawater is calculated by multiplying the sea-level freshwater saturation by^54^

4 $$ \omega_{s} = e^{{ - S\left( {1.7674 \times 10^{ - 2} + \frac{10.754}{{T_{abs} }} - \frac{2140.7}{{T_{abs}^{2} }}} \right)}} $$

#### Elevation, *k* (km)

The influence of atmospheric pressure on gas saturation at elevation is based on the standard atmosphere as described by the cubic polynomial^54^

5 $$ \omega_{k} = 1 - 0.11988 k + 6.10834 \times 10^{ - 3} k^{2} - 1.60747 \times 10^{ - 4} k^{3} $$

Additional insight into DO can be obtained by computing the rate of change of saturation by differentiating Eq. (2) with respect to temperature. Although functions like Eq. (2) can sometimes be differentiated analytically, the results are cumbersome and typically provide no insight. Numerical differentiation provides an alternative means to obtain the same results with the centered divided difference^55^

6 $$ h^{\prime}\left( x \right) = \frac{{h\left( {x + \lambda } \right) - h\left( {x - \lambda } \right)}}{2\lambda } $$

where *x* = the value of the independent variable, $h^{\prime}\left( x \right)$ = the function’s first derivative with respect to *x* evaluated at *x*, and $\lambda$ = a very small perturbation of *x*. For the present case, with *x* = *T* and $h\left( x \right) = o_{s} \left( T \right)$, the result is $do_{s} \left( T \right)/dT$ with units of (mgO_2_/L)/°C.

## Study area and data setting

### Study area

For this study, seven majorly polluted catchments of India^46,47^ were selected to analyze climate change impacts on DO with respect to RWT with various physiographic features. The seven river gauging stations are situated in India and are shown in Fig. 1a, and their main characteristics with study periods are outlined in Fig. 1b in tabular form. Two data sources were used to compile the models, with one being global, and one regional. We have used the Global Freshwater Quality Database (GEMSTAT) data for Narmada, Cauvery, Sabarmati, and Godavari catchments. We have used the Central Water Commission (CWC), India data for Tunga-Bhadra, Musi, and Ganga catchments. The Global Freshwater Quality Database GEMStat^56^ is hosted by the International Centre for Water Resources and Global Change (ICWRGC) and provides inland water quality data within the framework of the GEMS/Water Programme of the United Nations Environment Programme (UNEP). Approximately 500 water quality parameters were available in the global GEMSTAT database, out of which water temperature was used in this study for Narmada, Cauvery, Sabarmati, Godavari catchments when compiling models. The gauging stations are run by the Central Water Commission (CWC), India, and measure water temperature (T_w_) over a period of time (monthly mean of ten samples)^57^. We observed that majority of the time series retrieved from the source datasets (GEMSTAT and CWC) are discontinuous. To build a kNN-LSTM model, a complete dataset is necessary. To build an entire data record, the na.interp() method in R's forecast library was utilized to interpolate the missing observations using the STL (Seasonal and Trend decomposition using Loess) decomposition^58^. The meteorological data used in this work are monthly minimum (T_min_), and maximum (T_max_) air temperatures. T_min_, T_max_ was available from the India Meteorological Department (IMD) data on a 1° Latitude × 1° Longitude grids spatial resolution from 1951 to 2018. We have spatially interpolated the AT observations to the RWT gauging locations using linear interpolation. We averaged T_min_ and T_max_ to get the monthly mean AT as widely used literature^59^. Figure 1b shows the catchment means for all variables.

This study used the subset of the National Aeronautics Space Administration (NASA) Earth Exchange Global Daily Downscaled Projections (NEX-GDDP) dataset to assess the impact of climate change on RWTs for seven catchments of India. The NEX-GDDP is made up of downscaled climate scenarios for the entire world produced from the General Circulation Model (GCM) runs undertaken as part of the Coupled Model Intercomparison Project Phase 5 (CMIP5) and spanning two of the four greenhouse gas emissions scenarios known as RCPs^60^. The ensemble mean of the NEX-GDDP dataset contains RCP 4.5 and RCP 8.5 downscaled projections from the 21 GCMs models and scenarios, and each climate projection has daily maximum temperature, minimum temperature, and precipitation for 1950 through 2100. The dataset has a spatial resolution of 0.25° (~ 25 km × 25 km). This study retrieved the daily T_min_ and T_max_ values, converted them into a monthly scale, and averaged them to obtain the monthly mean AT for future RWT predictions.

### Data pre-processing

The applied data pre-processing consists of aggregating multiple data sources and feature engineering. We examined the data's autocorrelation and partial autocorrelation functions (ACF and PACF) to account for the time-lag information in RWT prediction at monthly time scale. These functions suggest that the 1-month time-lag is significant in the observed record. Thus, air temperature (AT[t]), and time-lag effects of air and water temperatures (AT[t − 1], RWT[t − 1]) are used as input variables in the prediction of RWT.

In the k-NN bootstrap resampling algorithm, “one simulation” is defined as a set of simulated values with a length equal to the observed dataset and chosen to generate 50 simulations. Following that, we ran a comparison study of monthly statistics (maximum, minimum, mean, standard deviation) for both the historical and simulated ensemble records. Also, we compared the lag-1 autocorrelation of the k-NN simulated data with observed data. The comparison has revealed that the algorithm produced the applicable distributional statistics of the observed dataset, implying that the algorithm generates accurate and diverse conditions. The lag-1 autocorrelation represents the relationship between two consecutive time steps (e.g., *x*_*t*_ and *x*_*t-1*_). When we compare the lag-1 autocorrelation of the historical and simulated record, we find that the lag-1 autocorrelation’s seasonality is frequently reproduced. Then, the simulated values of the monthly average ATs and RWTs were then used as model input in the LSTM model. In this study, while training a kNN-LSTM model on a time series, all the possible combinations of LSTM hyperparameter sets (the number of LSTM hidden layers: 1–3, the total number of units per layer: 5–100, time steps:1–12, the dropout ratio: 0–0.4, epochs: 50–100, and the batch size: 2–64) are evaluated using an emerging state-of-the-art Bayesian Optimization approach to optimize the hyperparameters, and the topmost group is chosen to improve the model’s performance.

## Results

The data used in this work comprises monthly average AT and the corresponding RWT for seven majorly polluted river locations in India. We used meteorological definitions of seasons: monsoon = June, July, August, September; post-monsoon = October, November; winter = December, January, February; and summer = March, April, May^61^. The catchment means of RWT, and AT for all seven catchments ranged between 24.68 °C, 30.34 °C, and 24.24 °C, 28.81 °C, respectively (Fig. 1b).

**Figure 2:**
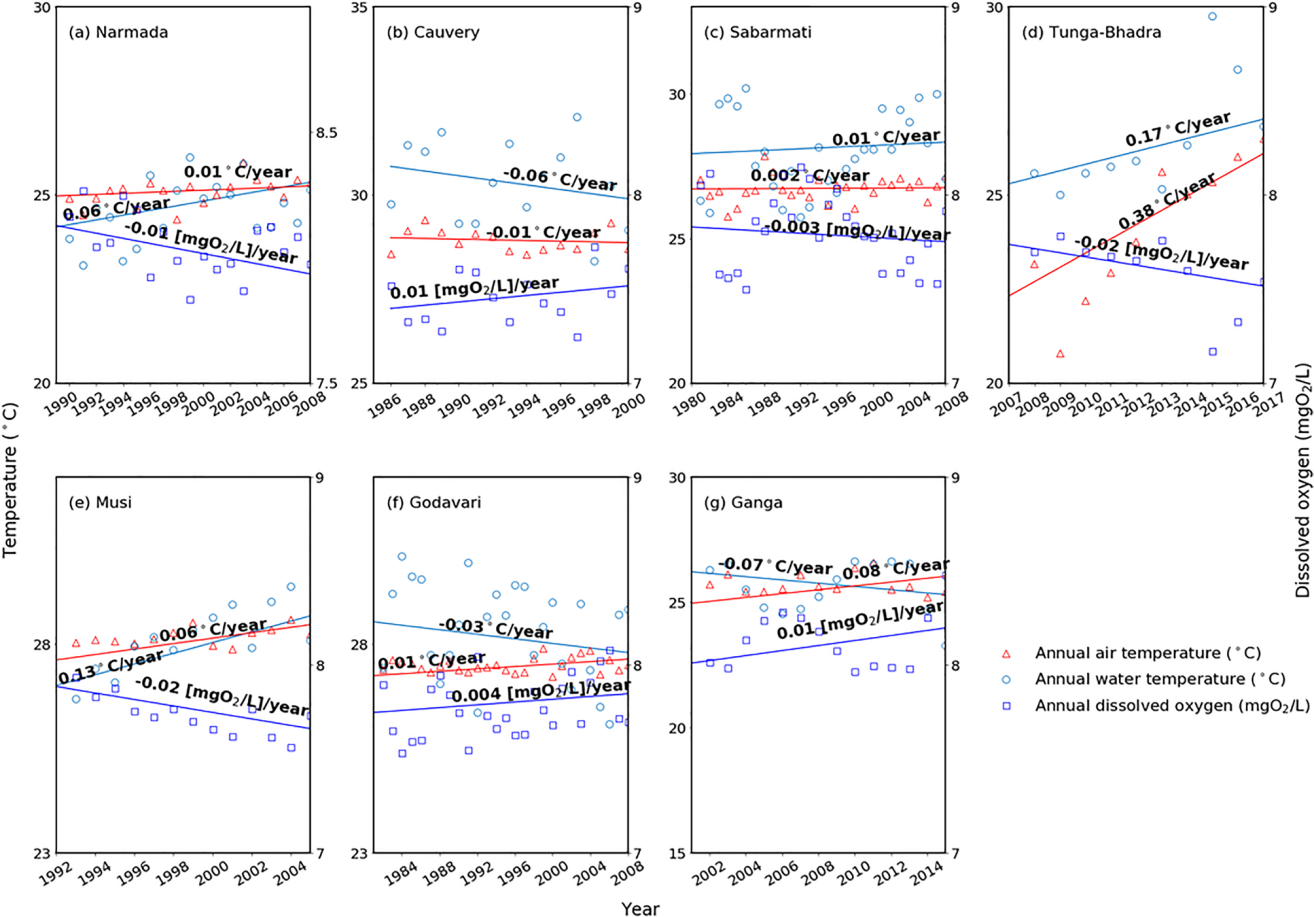
Seasonal, temporal variations of the mean annual air temperature (red), water temperature (light blue), and dissolved oxygen (blue) of the seven catchment stations (**a**) Narmada, (**b**) Cauvery, (**c**) Sabarmati, (**d**) Tunga-Bhadra, (**e**) Musi, (**f**) Godavari and (**g**) Ganga. Linear regressions of the time series are represented by trend lines, and the slope parameters are trend estimations.

To examine the variability of annually averaged AT, RWT, and DO changes, the study calculated the linear trends using the observed data for seven catchments of India (Fig. 2). The AT and RWT increased and observed DO has decreased during the studied period for all catchments except Cauvery, Godavari, and Ganga catchments (Fig. 2). The RWT rising rates are lower than those of AT in general.

Air temperature has shown a rising trend except for Cauvery (− 0.01 °C/year) catchment, and the rising rates range from 0.002 to 0.380 °C/year. RWT shows a rising trend except for Cauvery (− 0.06 °C/year), Godavari (− 0.03 °C/year), and Ganga (− 0.07 °C/year) catchments, and the rising rates vary between 0.01 and 0.17 °C/year. Such RWT rising patterns have been explored in several locations throughout the world. The RWT, for instance, has been a rising trend varying between 0.009 and 0.077 °C year^–1^ over the USA^3,62,63^, over China of about 0.029–0.046 °C year^–1^ ^64^, British Columbia as ~ 0.036 °C year^–1^ ^65^, and Europe as 0.006–0.180 °C year^–1^ ^66,67^.

DO shows a decreasing trend except for Cauvery (0.01 (mgO_2_/L)/year), Godavari (0.004 (mgO_2_/L)/year), and Ganga (0.01 (mgO_2_/L)/year) catchments (where there is a significant decreasing trend of AT and RWT has been noted), and the decreasing rates vary between − 0.01 and − 0.003 (mgO_2_/L)/year. Such DO decrease patterns have been explored in several locations throughout the world. The DO, for instance, has been a seasonal DO variation, low (DO < 10 mgO_2_/L) and high (DO > 14 mgO_2_/L) over Clackamas River near Oregon City, OR, USA^14^, and rising RWTs in the Delaware River, the USA by 2 °C to peak summer levels of 30 °C, based on saturation, DO levels will decline by about 0.2 mgO_2_/L^13^. Generally, RWT and AT are directly correlated, but RWT and DO are inversely correlated^14,16^. However, for the Godavari and Ganga catchment, the water temperature has shown decreasing trend (− 0.03 °C/year and − 0.07 °C/year respectively) with an increasing trend of AT (0.01 °C/year and 0.08 °C/year, respectively), which specifies that the temporal shifts of RWT may not be explained AT alone. RWT is directly influenced by multiple parameters, including streamflow^68^, river geometry, groundwater inputs, slope, water depth, etc.^69^.

### Deep learning model performance

**Figure 3:**
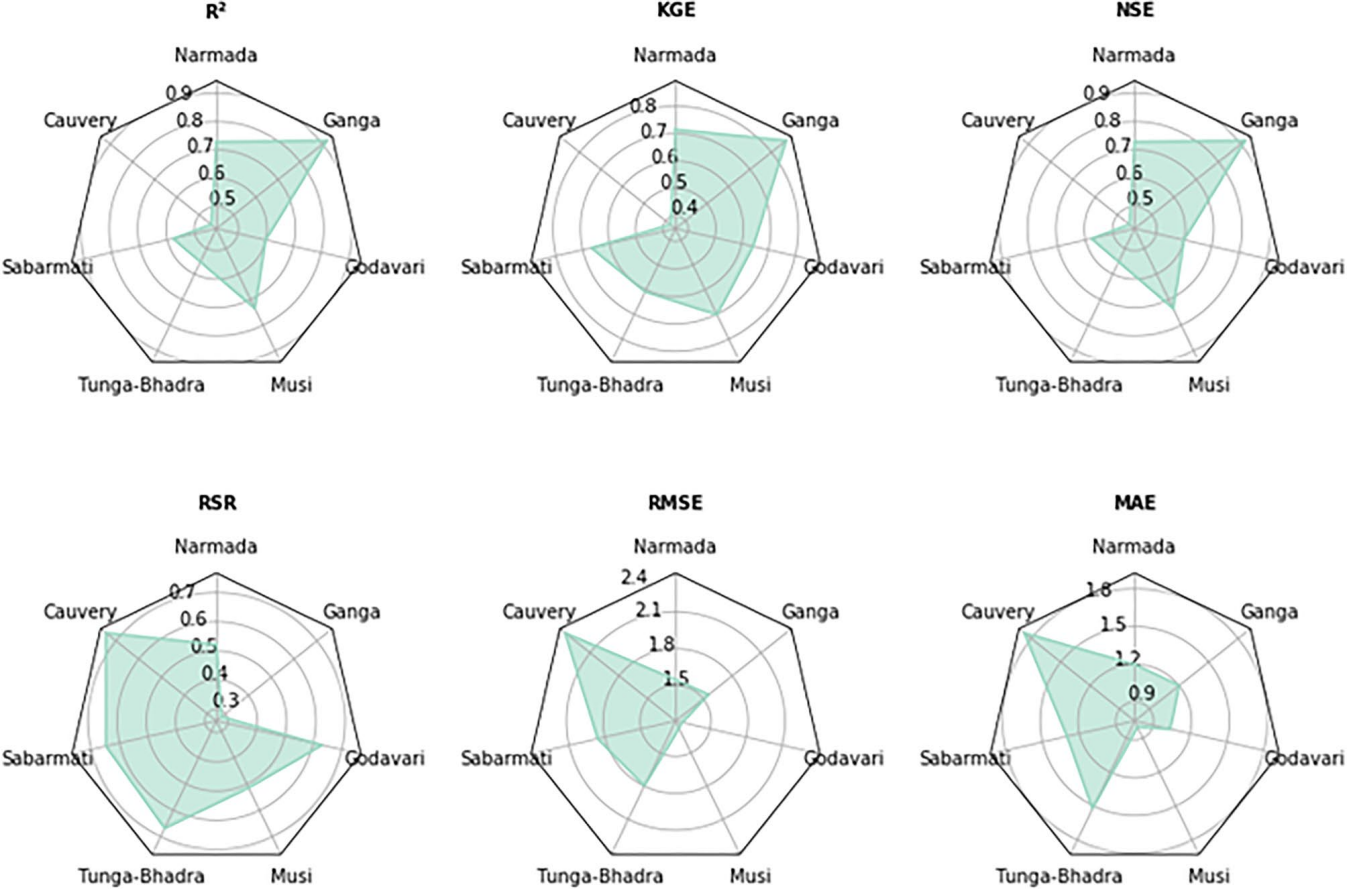
The representation of R^2^, KGE, NSE, RSR, RMSE, and MAE values in the form of a Radar plot for seven catchments for the kNN-LSTM model during the testing period.

The simulated samples from the k-NN bootstrap resampling algorithm and lag variables as input to the kNN-LSTM hybrid model to predict the RWT for monthly data for all the seven catchments of India. To mathematically quantify the predictive performances of kNN-LSTM approach, six statistical measures are calculated, such as the coefficient of determination (R^2^), Kling–Gupta efficiency (KGE)^70^, RMSE-observations standard deviation ratio (RSR)^71^, the root mean squared error (RMSE), Nash–Sutcliffe efficiency (NSE)^72^, and the mean absolute error (MAE) (Fig. 3). Detailed descriptions of these metrics can be found in Rajesh et al.^35^.

The relationship between monthly RWT and AT at seven catchments is relatively strongly correlated for the kNN-LSTM model (R^2^ and NSE values). The RMSE metrics varied from 1.266 to 2.361 for kNN-LSTM monthly data estimated between observed and simulated for all the catchments (Fig. 3). The NSE values for all the catchments range from 0.446 to 0.920 (Fig. 3) for the kNN-LSTM model for monthly data, which is reasonable compared with earlier standalone LSTM models by Stajkowski et al.^73^ (NSE: 0.913) and Qiu et al.^74^ (NSE: 0.74–0.99 °C). However, Stajkowski et al.^73^ used AT values as input for hourly data in their analysis, Qiu et al.^74^ used AT and discharge as input for daily data in RWT predictions, and the current study is dedicated to monthly timescales. Based on RSR, KGE, R^2^, and NSE performance values (Fig. 3), the kNN-LSTM model is the best performant model for all catchments. Overall, the kNN-LSTM model statistical metrics are reasonably within the range for all the catchment locations providing confidence that the developed model performs effectively.

The following analyses concentrate on how RWT affects the oxygen saturation of seven catchments of India.

### Oxygen saturation and oxygen concentration

**Figure 4:**
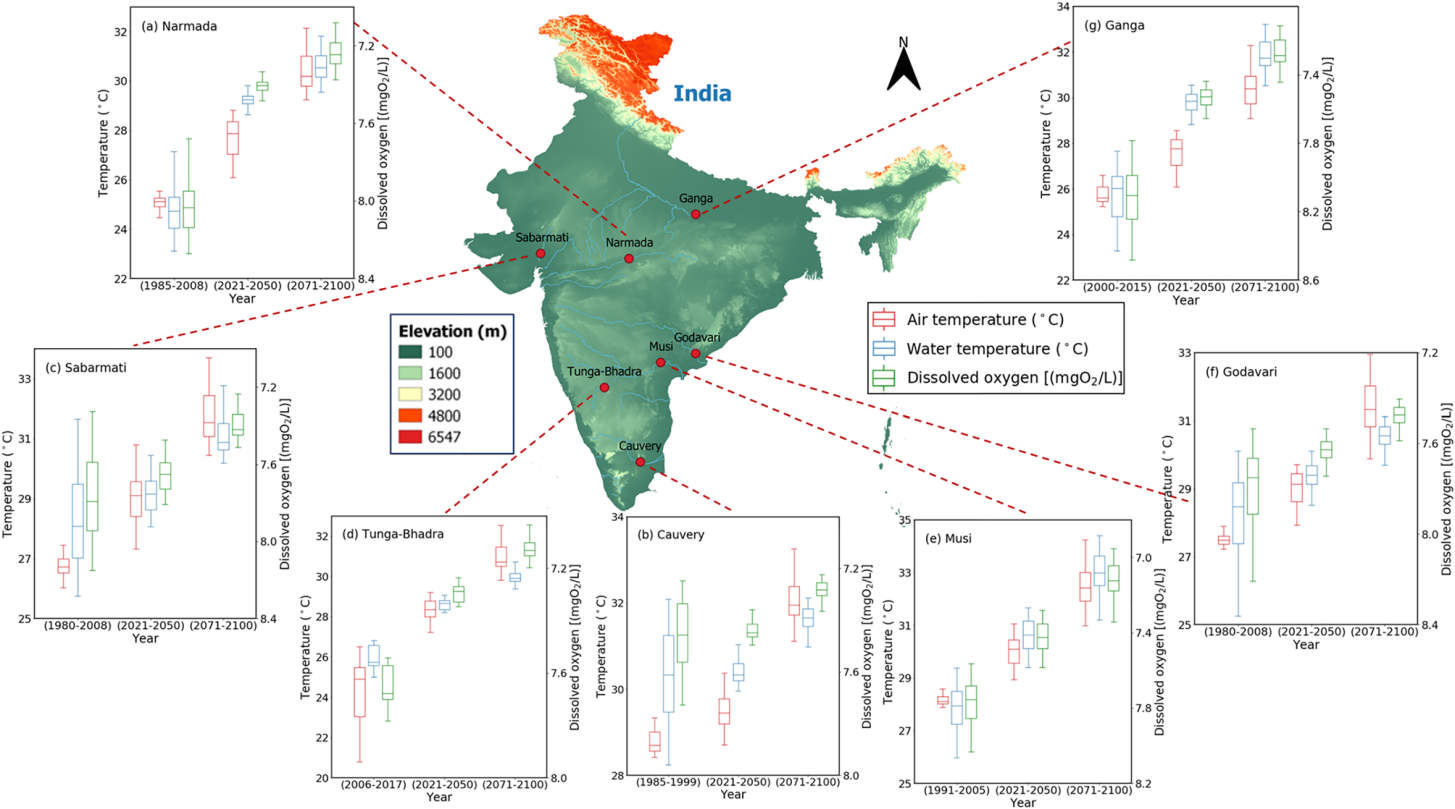
Boxplots represent the Representative Concentration Pathway (RCP) 8.5 experiments air temperature (°C) values; projected water temperature (°C) and dissolved oxygen (mgO_2_/L) values of historical, 2021–2050, and 2071–2100 for seven Indian catchments. The map was created using QGIS v3.4.14 (https://qgis.org), Python v3.7.4 (https://www.python.org), and post-processed with PowerPoint v2018 (https://microsoft.com).

**Figure 5:**
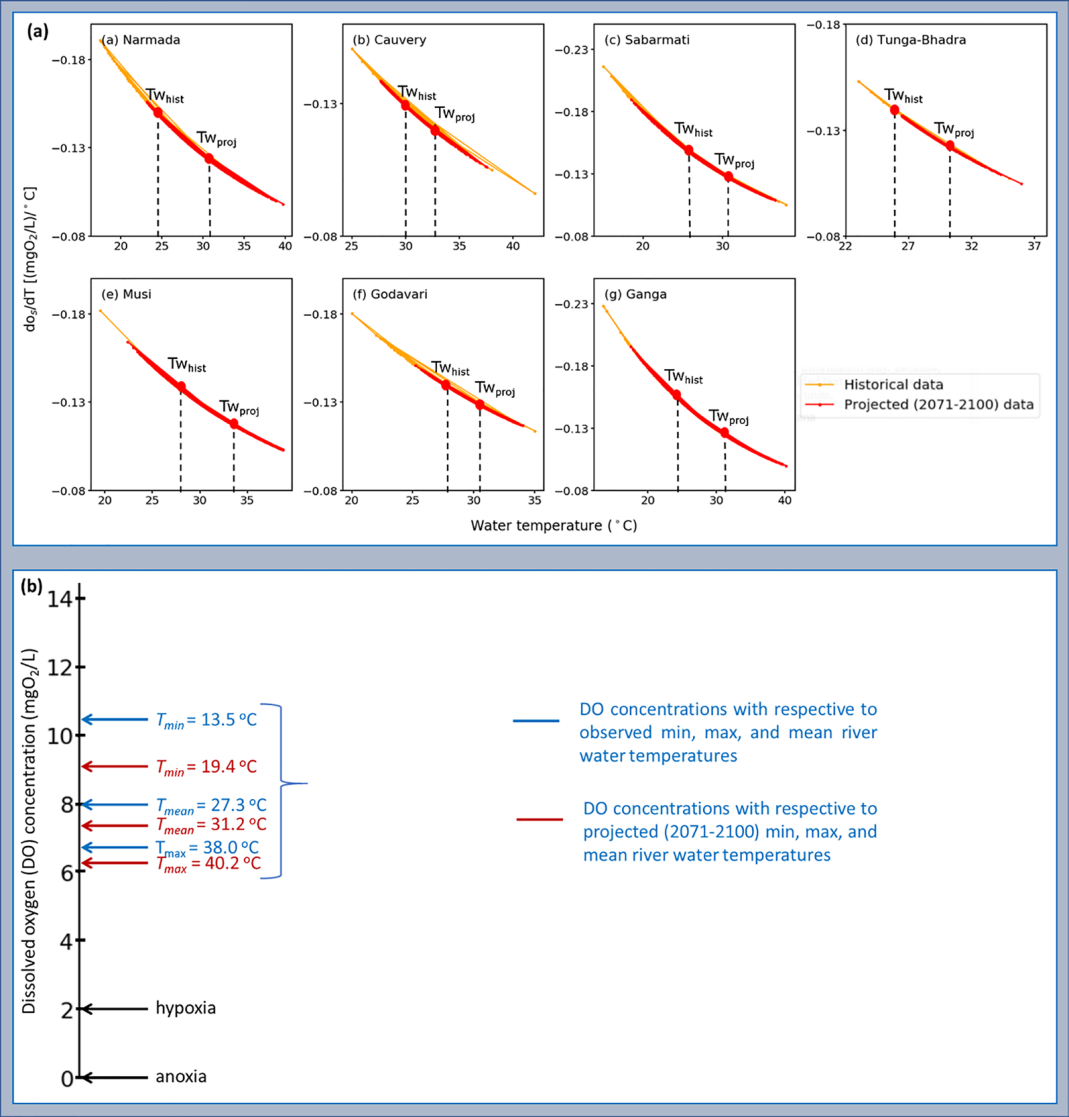
(**a**) The rate of change of oxygen saturation under mean river water temperature $d{o}_{s}(T)/dT$ ((mgO_2_/L)/°C) for historical and projected (2071–2100) data for seven Indian catchments. The vertical dotted lines indicate the mean of historical (Tw_hist_ °C) and projected (2071–2100) (Tw_proj_ °C) water temperatures, and (**b**) the DO concentration (mgO_2_/L) scale with respect to the observed (blue color) and projected (2071–2100) (red color) minimum, maximum and mean water temperature (°C) levels of seven Indian catchments.

Figure 4 displays the box plots of RCP 8.5 experiments air temperature (°C) values; projected RWT (°C) and DO (mgO_2_/L) values of historical, 2021–2050, and 2071–2100 for seven catchments of India. According to Fig. 4, due to the increase of AT, the saturated DO concentrations are decreased mainly due to the increases of RWT for the periods 2021–2050 and 2071–2100. Table 1 listed the rate of change of DO saturation levels under minimum, maximum, and average river water temperatures $d{o}_{s}(T)/dT$ ((mgO_2_/L)/°C) for observed and projected (2071–2100) for seven Indian catchments. Projected mean RWT changes for the periods 2071–2100 relative to mean observed values were calculated using RCP 8.5 output data and observed that results vary between the different catchments. The magnitude of DO decrease with respect to average RWT increase is higher for Narmada, Musi, and Ganga catchments, and variations in the rate of change of oxygen saturation for 2071–2100 relative to the historical values were noted as a drop of about 0.024, 0.018, and 0.025 (mgO_2_/L)/°C, respectively (Table 1). Moderate DO decreases with respect to mean RWT for 2071–2100 are projected for catchments in the southern parts of India relative to the historical values, noted as a drop of about 0.005 (mgO_2_/L)/°C for Cauvery and 0.009 (mgO_2_/L)/°C for Godavari (Table 1). The magnitude of DO decrease with respect to minimum RWT increase is higher for Narmada, Sabarmati, Godavari, and Ganga catchments, and with respect to maximum RWT increase is higher for Narmada, Musi, and Ganga catchments (Table 1). Overall, results indicated that DO with respect to RWT over Indian catchments would likely drop by more than 0.02 (mgO_2_/L)/°C for 2071–2100 (Table 1). Figure 5a shows the rate of change of DO saturation levels under mean river water temperature $d{o}_{s}(T)/dT$ ((mgO_2_/L)/°C) for observed and projected (2071–2100) data for seven Indian catchments. The vertical dotted lines indicate the mean of historical (Tw_hist_ °C) and projected (2071–2100) (Tw_proj_ °C) water temperatures. As depicted in Fig. 5a, projected (2071–2100) (Tw_proj_ °C) water temperatures increase is higher for Narmada, Tunga-Bhadra, Musi, and Ganga catchments compared to historical (Tw_hist_ °C), which leads to a higher drop in the rate of change of oxygen saturation for these catchments. For Cauvery catchment, projected (2071–2100) (Tw_proj_ °C) water temperatures increase is low compared to historical (Tw_hist_ °C), which leads to a minimal drop in the rate of change of oxygen saturation.

**Table 1:** The rate of change of oxygen saturation levels under a minimum, maximum, and average river water temperatures (in parentheses). ($do_{s} \left( T \right)/dT$ ((mgO_2_/L)/°C)) for historical and projected (2071–2100) at respective elevations for seven Indian catchments. Set the Salinity (*S*) value for seven river catchments to zero.

Catchment	Elevation (*k)* in km	Historical data			Projected data (2071–2100)			$do_{s} \left( T \right)/dT$ variation		
		$do_{s} \left( T \right)/dT$ (Tw_min_ (°C)) (1)	(Tw_max_ (°C)) (2)	$do_{s} \left( T \right)/dT$ (Tw_mean_ (°C)) (3)	$do_{s} \left( T \right)/dT$ ((Tw_min_ °C)) (4)	$do_{s} \left( T \right)/dT$ (Tw_max_ (°C)) (5)	$do_{s} \left( T \right)/dT$ (Tw_mean_ (°C)) (6)	(1)–(4)	(2)–(5)	(3)–(6)
Narmada	0.30	− 0.191 (17.5)	− 0.110 (35.0)	− 0.148 (24.7)	− 0.156 (23.2)	− 0.098 (39.8)	− 0.124 (30.6)	− 0.035	− 0.012	− 0.024
Cauvery	0.08	− 0.151 (25.0)	− 0.103 (38.0)	− 0.128 (30.4)	− 0.139 (27.7)	− 0.106 (37.5)	− 0.123 (31.7)	− 0.012	− 0.003	− 0.005
Sabarmati	0.05	− 0.216 (15.0)	− 0.105 (38.0)	− 0.137 (28.1)	− 0.160 (23.4)	− 0.109 (36.6)	− 0.126 (31.1)	− 0.056	0.004	− 0.011
Tunga-Bhadra	0.50	− 0.153 (23.0)	− 0.107 (35.0)	− 0.137 (26.4)	− 0.137 (26.5)	− 0.105 (35.9)	− 0.123 (30.0)	− 0.016	− 0.002	− 0.014
Musi	0.09	− 0.182 (19.5)	− 0.113 (35.0)	− 0.137 (27.9)	− 0.164 (22.4)	− 0.103 (38.6)	− 0.119 (33.0)	− 0.018	− 0.010	− 0.018
Godavari	0.02	− 0.180 (20.0)	− 0.114 (35.0)	− 0.137 (28.2)	− 0.148 (25.8)	− 0.117 (34.0)	− 0.128 (30.5)	− 0.032	0.003	− 0.009
Ganga	0.10	− 0.228 (13.5)	− 0.113 (34.9)	− 0.148 (25.6)	− 0.182 (19.4)	− 0.100 (40.2)	− 0.123 (31.8)	− 0.046	− 0.013	− 0.025

The specification of a DO water-quality standard, *o*_*wq*_ (mgO_2_/L), is used to evaluate oxygen assimilative capacity. Figure 5b shows the DO concentration (mgO_2_/L) scale with respect to the observed (blue color) and projected (2071–2100) (red color) minimum, maximum and average water temperature (°C) levels of seven Indian catchments. From Fig. 5b, observed that 10.3, 6.6, and 7.9 mgO_2_/L, and 9.1, 6.3, and 7.3 mgO_2_/L DO concentrations with respect to historical and projected (2071–2100) minimum, maximum and average water temperatures (°C) respectively of seven Indian catchments. DO concentration (mgO_2_/L) scale scores are dropped from 7.9 to 7.3 mgO_2_/L respective to the observed and projected (2071–2100) mean RWT levels of seven catchments (Fig. 5b). Table 2 listed the DO concentrations and DO decrease percentage with respect to monthly average summer and winter RWTs for historical and projected (2071–2100) with RCP 8.5 experiments for seven Indian catchments. The summer RWT increase for Tunga-Bhadra, Sabarmati, Musi, and Ganga basins are predicted as 3.1, 3.8, 5.8, 7.3 °C, respectively, with a more pronounced increase of 7.8 °C for the Narmada River for 2071–2100. The magnitude of DO concentrations decreases with respect to summer RWT increases is higher for Narmada, Musi, and Ganga catchment sites, and the percentage of DO decreases for 2071–2100 relative to the historical values noted 12.4, 9.3, and 11.9%, respectively (Table 2). The low DO concentrations decrease was observed for Cauvery and Godavari, and the percentage of DO decrease was noted as 1.0 and 3.3%, respectively (Table 2). Overall, the summer displayed larger percent decreases in DO compared to the winter season, and the largest DO decreases were found in the Narmada catchment (Table 2).

**Table 2:** The DO concentrations and percentage of DO decrease with respect to monthly average summer and winter (in parentheses) water temperatures for historical and projected (2071–2100) with Representative Concentration Pathway (RCP) 8.5 experiments for seven Indian catchments.

Catchment	Historical data		Projected data (2071–2100)		RWT (°C increase)	DO (%decrease)
	Tw_mean_ (°C)	DO (mgO_2_/L)	Tw_mean_ (°C)	DO (mgO_2_/L)		
Narmada	26.14 (22.42)	7.80 (8.37)	33.90 (27.08)	6.83 (7.68)	7.76 (4.66)	12.44 (8.24)
Cauvery	32.19 (29.78)	7.22 (7.52)	32.68 (30.43)	7.15 (7.43)	0.49 (0.65)	0.97 (1.20)
Sabarmati	28.60 (24.85)	7.70 (8.24)	32.39 (27.13)	7.21 (7.90)	3.79 (2.28)	6.36 (4.13)
Tunga-Bhadra	27.60 (25.78)	7.43 (7.67)	30.66 (28.62)	7.04 (7.29)	3.06 (2.84)	5.25 (4.95)
Musi	29.03 (25.82)	7.60 (8.05)	34.80 (29.37)	6.89 (7.56)	5.77 (3.55)	9.34 (6.09)
Godavari	29.12 (26.59)	7.66 (8.01)	30.94 (27.88)	7.41 (7.82)	1.82 (1.29)	3.26 (2.37)
Ganga	25.04 (19.44)	8.15 (9.09)	32.34 (26.40)	7.18 (7.96)	7.30 (6.96)	11.90 (12.43)

## Discussion

This study presents new intuitions on the assessment of climate change impacts on saturated DO concentrations with respect to RWT for seven different catchment sites across India in different physiographic settings. For this, using the monthly kNN-LSTM prediction model, which is developed based on AT, including time-lag effects, demonstrates rising RWTs will reduce a river's assimilative capacity by affecting its oxygen metabolism, in addition to lowering saturation.

The monthly R^2^ scores estimated between observed and simulated RWT using kNN-LSTM for various stations ranged between 0.446 and 0.920, KGE scores ranged between 0.378 and 0.868, NSE scores ranged between 0.446 and 0.920, RSR scores ranged between 0.283 and 0.744, and RMSE scores were ≤ 2.4 °C during the testing periods for kNN-LSTM prediction model, revealing high model reliability. All the developed model statistical metrics covered the range of model reliability described in the literature. The RMSE scores for all the catchments ranged between 1.266 and 2.361 °C pertaining to the kNN-LSTM model for monthly data, which are reasonable in comparison to earlier models of the Spatio-temporal approach by Jackson et al.^75^ (1.570 °C); Bayesian regression approach by Sohrabi et al.^68^ (1.250 °C); random forest (RF), ANN, recurrent neural networks (RNNs) by Feigl et al.^76^ (0.422–0.815 °C); extreme gradient boosted tree algorithm and support vector regression by Weierbach et al.^36^ (0.92–1.02 °C); Wavelets-ANN by Graf et al.^77^ (0.981–1.434 °C); LSTM by Stajkowski et al.^73^ (0.755 °C); LSTM by Qiu et al.^74^ (0.500–2.700 °C); and ANN by Temizyurek et al.^78^ (2.100–2.640 °C). The MAE values for all the catchments range from 0.802 to 1.872 °C pertaining to the kNN-LSTM model for monthly data, which are reasonable in comparison to earlier models of RF, ANN, RNN by Feigl et al.^76^ (0.329–0.675 °C); Wavelets-ANN by Graf et al.^77^ (0.781–1.286 °C); and LSTM by Qiu et al.^74^ (0.39–2.15 °C). The KGE values for Narmada (0.715), Tunga-Bhadra (0.790), Musi (0.701), and Ganga (0.868) catchments pertaining to the kNN-LSTM model for monthly data, which are reasonable compared to the earlier model of LSTM by Stajkowski et al.^73^ (0.923). The NSE values for Narmada (0.728), Musi (0.735), and Ganga (0.920) catchments pertaining to the kNN-LSTM model for monthly data, which are sensible compared to the earlier model of LSTM by Qiu et al.^74^ (0.74–0.99). The superiority of LSTM in RWT prediction, as demonstrated in this work, was found to agree with Feigl et al., Qiu et al., and Stajkowski et al.^73,74,76^. However, it can be noted that the study was conducted by Feigl et al.^76^ used AT, runoff, precipitation, and global radiation values as input in the RWT prediction for daily data, the study by Qiu et al.^74^ used daily AT, and discharge as input in RWT prediction, and the study by Stajkowski et al.^73^ used AT values as input in RWT prediction for hourly data.

The RWT increases of up to7 °C for summer, reaching close to 35 °C, decreases DO by 2–12%, thus decreasing the saturation capacity for DO for 2071–2100. DO concentration (mgO_2_/L) scale scores are dropped from 7.9 to 7.3 mgO_2_/L respective to the observed and projected (2071–2100) mean RWT levels of seven catchments. These scores reveal that DO concentration (mgO_2_/L) values are dropping for projected years as RWTs rise. The RWT increases of up to7 °C for summer, demonstrated in this work, were found to agree with Chapra et al.^1^ (5 °C increments in summer RWTs in most of the world’s rivers over the next 50 years). The DO concentration (mgO_2_/L) scale scores, as demonstrated in this work, were found to agree with Du et al.^15^ (DO concentrations on the basin average scale will decrease by 0.72 mgO_2_/L under RCP 8.5 scenario for 2061–2100) and Chapra et al.^1^ (DO oxygen concentrations are 9.0 and 6.8 mgO_2_/L for freshwater temperatures 20 and 35 °C, respectively).

The percentage of DO decrease with respect to summer RWTs is higher for Narmada, Musi, and Ganga catchment sites for 2071–2100 relative to the historical values noted as 12.4, 9.3, and 11.9%, respectively, probably because of the influence of disposal of untreated sewage and industrial wastewater along with due to increased reaction kinetics at a higher temperature under climate change scenarios (Table 2). In this study, overall, for all seven catchments, the decrease in DO is 8% for the plausible future (2071–2100) (Fig. 5b and Table 2). These projected change patterns are most consistent with earlier hydrological model studies by Ficklin et al.^16^ (10% decreases in DO by 2100 at Sierra Nevada in California, USA) and by Du et al.^15^ (DO decrease on the basin average scale by 0.72 mgO_2_/L under RCP 8.5 scenario for 2061–2100 in the Athabasca River Basin, Canada). Overall, this study demonstrated how river oxygen levels would be influenced by rising RWT due to climate change using the kNN-LSTM model for the Indian riverine system. The rising RWTs will reduce river assimilative capacity by affecting its oxygen metabolism, in addition to lowering saturation, and necessitates redefining/alterations of the river water quality standards under climate change.

Furthermore, the DO simulated by Eq. (2) is the saturated oxygen concentration, which is the total amount of DO that can be dissolved within the streamflow volume, and thus, it can be expected that the DO concentrations presented in this study represent the ceiling of potential DO levels. Though the hybrid kNN-LSTM model performed well, further research is needed to improve it. We found that inherent uncertainties from the kNN-LSTM model can accumulate and affect the final performance measurements. Such uncertainties can originate from various sources, from noise and temporal discontinuity present in the original water quality sampled observations to the model hyperparameters used to predict RWT. To address such model uncertainties, the ensemble of DL models, etc.^79,80^ can be adopted which can combine RWT predictions from various DL models, allowing the decision-makers to choose the best possible prediction within a range of predictions^81^. Despite the effectiveness of the modeling frameworks, as demonstrated in the present work, it has some limitations. Firstly, flow discharge may play a vital role in RWT predictions, especially the Indian rivers are significantly impacted by low flows in the summer season. However, flow discharge was not examined in this work due to a lack of complete data. Therefore, we will strengthen the hybrid modeling framework in future research by integrating flow discharge as model input for rivers. Secondly, RWT is directly influenced by multiple parameters, including streamflow^28,65,68^, river geometry, groundwater inputs, slope, water depth, etc.^69^, which are not considered in the present study. This study set the Salinity (S) value for seven river catchments to zero because most rivers and streams had minimal salinity^1^. Overall, this research offers vital intuitions about the historical and projected RWT and DO states of major Indian river catchment locations, which may be beneficial in creating future water management plans that may impact aquatic resources.

## Conclusions

The study demonstrates the climate change impacts on saturated DO concentrations with respect to RWT for the seven major polluted Indian catchments at a monthly timescale. The hybrid kNN-LSTM model is implemented in this study to predict the RWT addressing sparse spatiotemporal RWT data. Further assessed the climate change impacts on DO concentrations with respect to RWT using a forced by an ensemble of RCP 8.5 scenario downscaled projections of AT data from the NEX-GDDP dataset. The results lead to the following conclusions:

1. An increase in AT will have an effect on RWTs, and saturated DO concentrations. The latter will trigger higher RWT and lower DO concentration. These changes appear especially significant for the summer seasons and include RWT increases of up to7 °C for summer, reaching close to 35 °C, decreases of DO by 2–12%, thus decreasing the saturation capacity for DO.
2. The percentage of decrease of DO saturation levels with respect to summer RWTs is higher for Narmada, Musi, and Ganga catchment sites for 2071–2100 relative to the historical values noted as 12.4, 9.3, and 11.9%, respectively.
3. DO concentration (mgO_2_/L) scale scores are dropped from 7.9 to 7.3 mgO_2_/L respective to the observed and projected (2071–2100) mean RWT levels of seven catchments.
4. Overall, saturated DO concentration (mgO_2_/L) levels are dropping by 8% under the rise of summer RWT by more than 4.3 °C for 2071–2100. That is, for every 1 °C RWT increase, there will be about 2.3% decrease in DO saturation level concentrations over Indian catchments under climate signals.
5. The study provides an assessment of the individual contribution of RWT rise on depletion of saturated DO levels, which is helpful for the policymakers and pollution control authorities for sustainable river water quality management.
